# Association between meteorological conditions and dizziness-related ambulance transports: a population-based study in Kochi Prefecture, Japan

**DOI:** 10.1265/ehpm.26-00085

**Published:** 2026-10-20

**Authors:** Marina Minami, Seiji Hirota, Atsushi Morizane, Natsuko Nakamura, Hiroaki Ito, Masamitsu Eitoku, Atsufumi Kawauchi, Narufumi Suganuma, Kingo Nishiyama, Masato Miyauchi

**Affiliations:** 1 Integrated Center for Advanced Medical Technologies (ICAM-Tech), Kochi Medical School Hospital, Kochi University, Nankoku, Kochi 783-8505, Japan; 2 Department of Anesthesiology and Intensive Care Medicine, Kochi Medical School, Kochi University, Nankoku, Kochi 783-8505, Japan; 3 Department of General Medicine, Kochi Medical School, Kochi University, Nankoku, Kochi 783-8505, Japan; 4 Department of Otolaryngology, Kochi Medical School, Kochi University, Nankoku, Kochi 783-8505, Japan; 5 Department of Environmental Medicine, Kochi Medical School, Kochi University, Nankoku, Kochi 783-8505, Japan; 6 Department of Health Policy, Kochi Prefectural Government, Kochi, Kochi 780-8570, Japan; 7 Department of Disaster and Emergency Medicine, Kochi Medical School, Kochi University, Nankoku, Kochi 783-8505, Japan

**Keywords:** Dizziness, Ambulance transport, Meteorological factors, Ambient temperature, Seasonality

## Abstract

**Background:**

Dizziness is a common but nonspecific symptom that frequently leads to emergency department visits and ambulance transports, particularly when symptoms are sudden or severe. Although meteorological conditions affect various acute health outcomes, we still know little of their associations with dizziness-related emergency transports. This study examined the relationship between short-term meteorological conditions and dizziness-related ambulance transports using population-based data.

**Methods:**

We conducted a retrospective population-based study using emergency transport record data from the Kochi-Iryo-Net database in Kochi Prefecture, Japan, between January 2017 and December 2023. Dizziness cases were identified based on physician-recorded diagnoses at the receiving medical institutions. Daily meteorological data were obtained from the Japan Meteorological Agency. The days were classified according to the number of dizziness-related ambulance transports (low-frequency days for dizziness-related ambulance transports, <5 per day vs. high-frequency days for dizziness-related ambulance transports, ≥5 per day). Logistic regression analyses were performed to examine the associations between meteorological conditions (e.g., mean air temperature, sea-level pressure, short-term temperature changes) and high-frequency days for dizziness-related ambulance transports.

**Results:**

In total, 270,120 eligible emergency transports were analyzed, including 8,981 dizziness-related cases. Dizziness-related ambulance transports tended to be more frequent during the summer months. A higher mean air temperature was consistently associated with increased odds of high-frequency days. Short-term increases in maximum temperature and smaller diurnal temperature ranges were also associated with high-frequency days, whereas the associations with sea-level pressure were weaker and less robust. The absolute differences in meteorological variables were modest.

**Conclusions:**

Higher ambient temperatures and short-term temperature-related changes were associated with increased odds of high-frequency days for dizziness-related ambulance transports. Meteorological conditions may thus be associated with temporal variations in dizziness-related emergency transport demands. Further studies are warranted to clarify the underlying mechanisms and specific causes of dizziness associated with these patterns.

**Supplementary information:**

The online version contains supplementary material available at https://doi.org/10.1265/ehpm.26-00085.

## Graphical Abstract

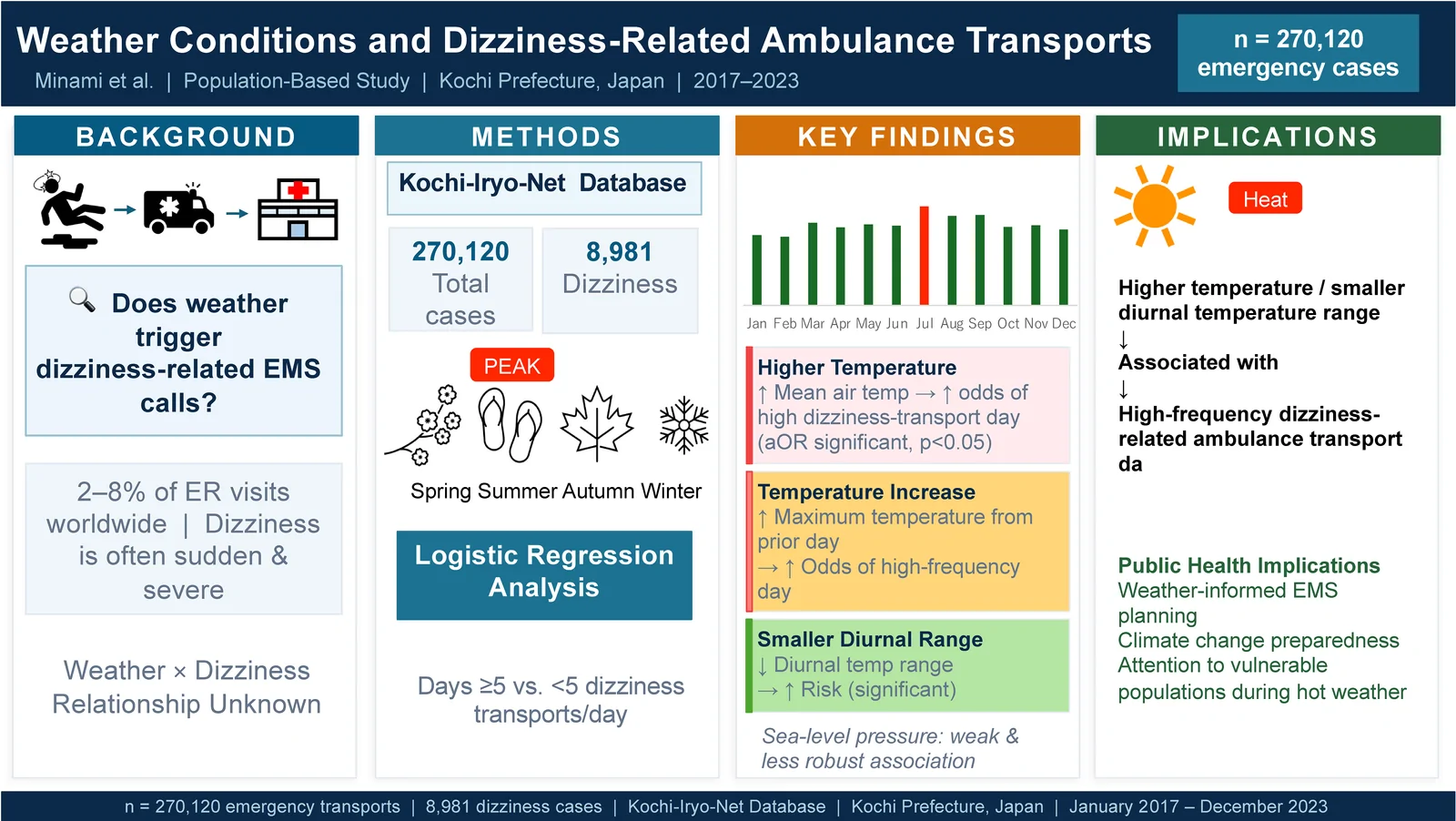

## Background

Dizziness is a very common but nonspecific symptom that affects up to one-third of people at least once during their lifetime, accounting for 2–8% of primary care and emergency visits [1, 2]. The term covers various sensations, including a spinning feeling (vertigo), imbalance, lightheadedness, or a vague sense of unsteadiness [1, 3]. The causes of dizziness are often classified into peripheral or central causes. Peripheral dizziness arises from disorders of the inner ear and vestibular nerve, such as benign paroxysmal positional vertigo, vestibular neuritis, and Ménière’s disease; while these conditions can cause short-term disabilities, they are usually benign [3–5]. Central dizziness results from brain problems, including stroke, demyelinating diseases, and tumors, usually necessitating urgent diagnosis and treatment because of the risk of permanent neurological damage or death [6, 7]. Because it is common for patients to struggle to describe the exact “quality” of their dizziness, modern approaches to dealing with the symptom emphasize timing and triggers (e.g., onset, duration, and provoking factors) rather than the patient’s reporting label (e.g., “vertigo” vs. “lightheadedness”) [3, 4, 8]. In the absence of major red flags, routine laboratory tests and brain imaging are often unnecessary and have low diagnostic yield [2, 9]. Moreover, the treatment depends on the underlying cause: benign paroxysmal positional vertigo is effectively managed with canalith repositioning maneuvers (e.g., the Epley maneuver); Ménière’s disease is treated with salt restriction and diuretics; vestibular neuritis with short-term vestibular suppressants and rehabilitation [3–5]. Importantly, vestibular rehabilitation is critical in improving balance and reducing chronic symptoms across etiologies [1, 4].

Although most dizziness cases are ultimately benign, a significant minority are associated with serious neurological diseases, making careful assessment essential to avoid overlooking potentially life-threatening conditions [9, 10]. Consequently, dizziness is a common reason for seeking emergency medical care, accounting for approximately 2–4% of all emergency department visits [11]. Furthermore, the need to distinguish benign from serious causes often requires substantial healthcare resources, including diagnostic imaging, prolonged emergency department evaluation, and hospital admission, resulting in a considerable burden on emergency medical services and healthcare systems [12, 13].

Recent studies show that air temperature, atmospheric pressure, sunshine duration, and precipitation are important human health determinants. For example, many epidemiological studies have reported a U-shaped relationship between temperature and mortality, with both heat and cold increasing cardiovascular and respiratory events, and long-term exposure to unusual temperatures raising chronic cardiopulmonary risks [14, 15]. Atmospheric pressure fluctuations or extremes can trigger acute problems (e.g., myocardial infarction, stroke, migraine, and other neurological or circulatory disorders), as well as modulate biological rhythms and metabolic markers [16]. Moreover, sunshine duration influences health both directly through thermal and ultraviolet exposure and indirectly by modifying air pollution levels, while varying sunshine and rainfall have been associated with trauma changes, psychiatric admissions, and infectious and respiratory disease patterns [16–20]. Indeed, precipitation and humidity shape the seasonality of many infections (e.g., influenza and diarrheal diseases), contribute to water- and vector-borne disease risks, and interact with temperature to amplify climate-sensitive diseases and extreme-weather health impacts [19, 20]. Overall, these meteorological conditions jointly affect infectious diseases along with cardiovascular, respiratory, and mental health, with older adults and socially vulnerable groups generally being at a higher risk of associated problems, highlighting the need for integrated weather- and climate-informed public-health planning [14, 19, 21].

Short-term weather conditions are also significantly associated with some acute health outcomes that require emergency transport. For instance, in a population-based analysis of emergency transport data from Kochi Prefecture, Japan, a lower temperature at onset was identified as a significant climatic factor influencing subarachnoid hemorrhage development [22]. Despite the growing evidence that meteorological factors influence various acute health events, their effects on dizziness and dizziness-related emergency transport remain poorly understood. Importantly, there has been a recent hospital-based study in Japan which examined the association between meteorological conditions and emergency transports for vertigo/dizziness, reporting significant correlations with temperature, humidity, and precipitation [23]. The problem is that this study was conducted at a single urban tertiary care center and may not fully reflect the characteristics of community-based populations, particularly in rural areas with a rapidly aging population. Additionally, to our knowledge, there are few population-based studies that have attempted to capture the real-world burden of dizziness-related emergency transports in regional settings. The same occurs for large-scale research examining whether short-term weather condition variations are associated with high-frequency days for dizziness-related ambulance transports. This study endeavored to address these gaps by using population-based data from Kochi Prefecture, Japan, to examine the association between short-term meteorological conditions and high-frequency days for dizziness-related ambulance transports.

## Methods

### Study design and setting

Japan has a nationwide, taxpayer-funded, combined fire and ambulance service. It is freely available to anyone, anytime, anywhere in the country through an emergency phone number (i.e., 119). Local governments provide ambulance services through their fire departments, while municipal governments oversee fire department management. Additionally, the national (i.e., Fire and Disaster Management Agency, the Ministry of Internal Affairs and Communications) and prefectural governments and agencies provide guidance and advice to municipal fire departments.

This study was conducted in Kochi City, the capital of Kochi Prefecture, located on Shikoku Island, southwestern Japan. Kochi Prefecture faces the Pacific Ocean and has a humid subtropical climate characterized by relatively warm temperatures and abundant rainfall throughout the year [24]. In 2023, 36.3% of the prefecture’s population was aged 65 years or older, making it one of the most rapidly aging regions in Japan [25, 26]. As of 2021, Kochi Prefecture had 15 fire departments. Kochi Prefecture features the Kochi-Iryo-Net database, which was established as part of its emergency medical and disaster information system; whenever a fire department dispatches an ambulance and its crew within the Prefecture, dispatch-related information (e.g., fire department, date, and time of call) is recorded in the Kochi-Iryo-Net database. Upon the patient’s arrival at the medical institution, nurses and the attending physician record information pertinent to the case (e.g., degree of severity and disease classification) in the institution’s system for medical record management purposes. This information is later aggregated into the Kochi-Iryo-Net database according to the destination medical institution’s data from the Kochi prefectural government. In this retrospective study, we used aggregated data from this database.

Specifically, the database was used to include patients transported by ambulance in the sample. As mentioned in the previous paragraph, after emergency transportation reaches the medical institution, the attending physician in charge enters information about the case in the institution’s system, having the possibility of entering up to three diagnoses based on a list of injuries available in the system. The dataset we used included all emergency calls recorded between January 1, 2017, and December 31, 2023, with each record containing information on call date and time, patient characteristics, and diagnoses as recorded by physicians at the receiving medical institutions. Cases were classified as dizziness-related when at least one of the three disease/injury name fields completed by the attending physician (following ambulance transport) at the receiving medical institution contained terms indicating dizziness, vertigo, or contained diagnoses such as benign paroxysmal positional vertigo, benign paroxysmal positional dizziness, and Ménière’s disease. Information regarding physician specialty, clinical department, and diagnostic procedures was not available in the database.

Considering the emergency transport records between January 1, 2017, and December 31, 2023, a total of 289,883 records were initially identified. Records with missing or ineligible information, including for age-, sex-, location-, or injury-related information, were excluded (n = 19,763). After these exclusions, 270,120 eligible emergency transport records were included. Among these, a total of 8,981 cases of dizziness were identified based on the physician-recorded diagnoses entered at the receiving medical institutions.

The eligible study period encompassed 2,556 days, which were categorized as high- or low-frequency days according to the daily number of dizziness cases and the operational cutoff described below. These day-level data were used for subsequent analyses (Fig. 1).

**Fig. 1:**
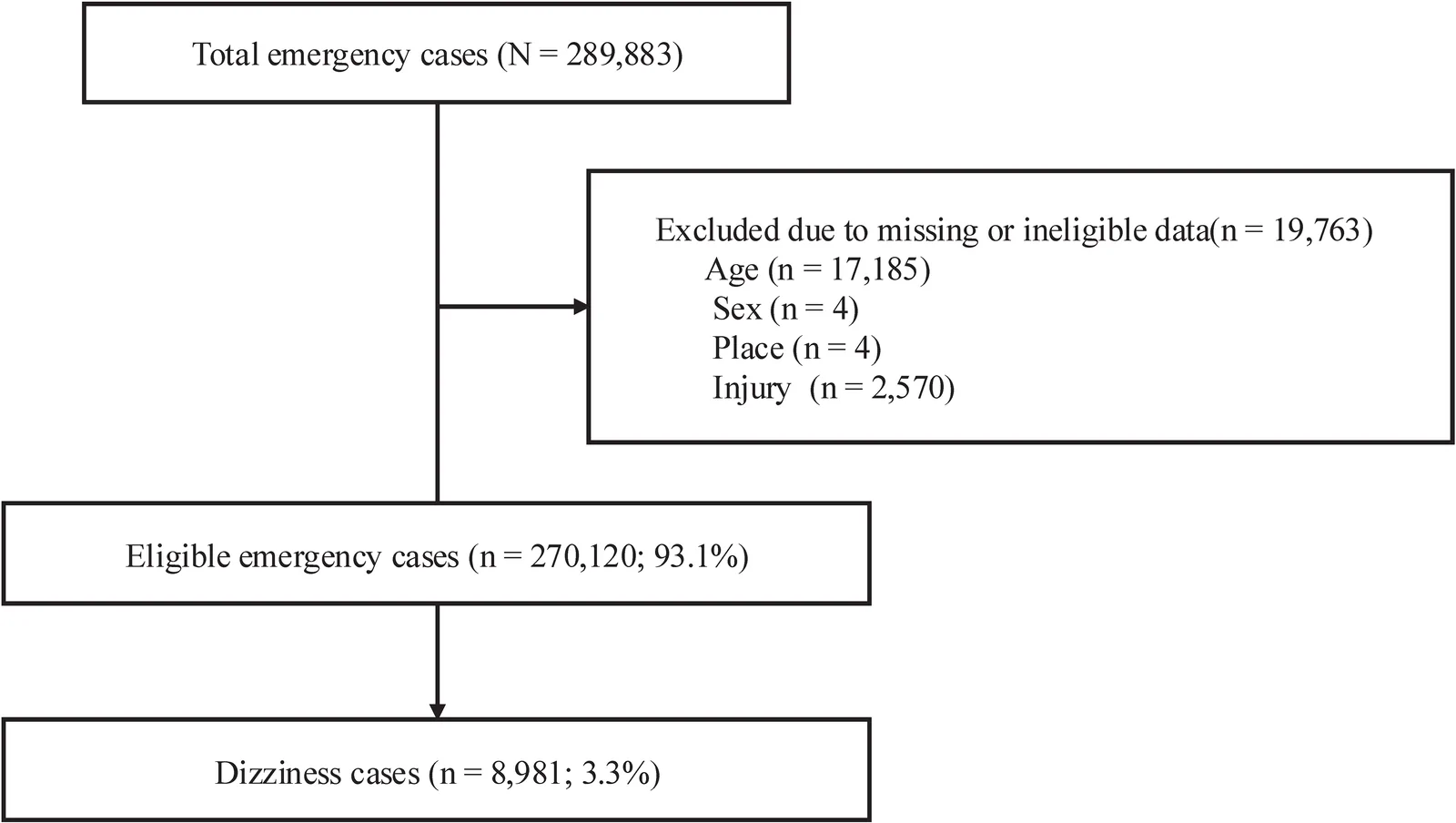
Study population flow diagram Among the 289,883 emergency ambulance transport cases recorded during the study period, 19,763 were excluded owing to missing or ineligible data (i.e., for age group, sex, place of occurrence, or injury severity). The final analytical sample comprised 270,120 eligible emergency cases, of which 8,981 (3.3%) were identified as dizziness-related ambulance transports.

To ensure patient confidentiality, we anonymized all data in accordance with the Kochi Prefecture’s medical policy, a process that was supervised by the Kochi prefectural government. The study protocol was approved by the Ethical Review Committee of the Kochi University School of Medicine (approval number # 2021-142; approval date: 11 January 2022) and adhered to the ethical standards outlined in the Declaration of Helsinki. Informed consent was waived because all data were anonymized prior to the analysis. In accordance with the requirements of the ethics committee, information regarding the study was publicly disclosed through an opt-out procedure, allowing individuals the opportunity to decline participation.

### Data collection and measurements

The primary outcome measure was the daily number of emergency ambulance transports related to dizziness, with dizziness-related ambulance transports being identified, as aforementioned, based on physician-recorded diagnoses entered at the receiving medical institutions after patient arrival.

For each emergency transport record, information on call date and time, patient age and sex, location of the dispatching fire station, and departure site were extracted from the database. Individual-level clinical details, such as comorbidities, medication use, and symptom duration, were not available in the dataset, and thus were not accounted for in the study.

Meteorological data were obtained from the Japan Meteorological Agency. Data from four observation stations within the study region with the most complete records during the study period were selected (Kochi, Tosashimizu, Sukumo, and Muroto) to ensure data completeness. Daily meteorological variables, including air temperature, sea-level pressure, precipitation, and sunshine duration, were extracted. Mean sea-level pressure was used as a proxy for atmospheric pressure because it is standardized for altitude and is commonly used in meteorological and epidemiological studies [27]. Each ambulance transport record was linked to the nearest meteorological observation station based on the location of the fire department that received the emergency call, and meteorological data were merged with ambulance transport records by date. These linked day-level data were used to assess the association between meteorological conditions and dizziness-related emergency ambulance transport.

### Data analysis

To explore seasonal variation, daily counts of dizziness-related ambulance transports were aggregated into monthly totals, and their distributions were descriptively assessed. Patient characteristics were summarized using counts and percentages for categorical variables. The baseline characteristics of emergency transports were compared between dizziness- and non-dizziness-related cases. Categorical variable differences, including in age group, sex, season, place of occurrence, injury severity, and one-week outcomes, were assessed using chi-square test.

To further evaluate the individual-level associations between meteorological conditions and dizziness-related ambulance transports, logistic regression analyses were performed using dizziness-related ambulance transport as the outcome variable. Univariable logistic regression analyses were first conducted for each meteorological variable. Subsequently, multivariable logistic regression analyses were performed adjusting for age group, sex, season, place of occurrence, and injury status, calculating crude (cORs) and adjusted odds ratios (aORs) with 95% confidence intervals (CIs). The multivariable models simultaneously included all meteorological variables shown in the tables.

For the day-level analyses, no established threshold exists, to our knowledge, for defining a high-frequency day for dizziness-related ambulance transports. Thus, the distribution of daily dizziness-related ambulance transports was examined, and the third quartile was used to establish a methodological cutoff. This approach was intended to ensure a sufficient sample size for statistical analysis while identifying days with a relatively high volume of transports. Based on this criterion, a cutoff of five or more transports per day was selected, classifying the study period into high-frequency days (≥5 dizziness-related ambulance transports; n = 701 days, representing 27.4% of the total days) and low-frequency days (<5 dizziness-related ambulance transports; n = 1,855 days). Sensitivity analyses were subsequently conducted using alternative thresholds around the primary cutoff.

Additional logistic regression analyses were performed to assess the associations between daily changes in meteorological variables and the occurrence of high-frequency days for dizziness-related ambulance transports. Daily changes were calculated as the value on a given day minus the corresponding value on the previous day. The variables examined included changes in maximum temperature, minimum temperature, diurnal temperature range, and mean sea-level pressure.

To assess potential multicollinearity among meteorological variables included in the multivariable models, pairwise correlation coefficients and variance inflation factors were examined. All statistical tests were two-sided, and a *p* < 0.05 was considered statistically significant. All analyses were performed using Stata/MP software (version 16.0; StataCorp).

## Results

Figure 2 illustrates the monthly distribution of dizziness-related ambulance transports. Ambulance transports tended to be more frequent during the summer months, particularly in July. This seasonal pattern motivated further analyses into the association between meteorological conditions and high-frequency days for dizziness-related ambulance transports (Fig. 2). The monthly distribution by study year is shown in Supplementary Fig. S1.

**Fig. 2:**
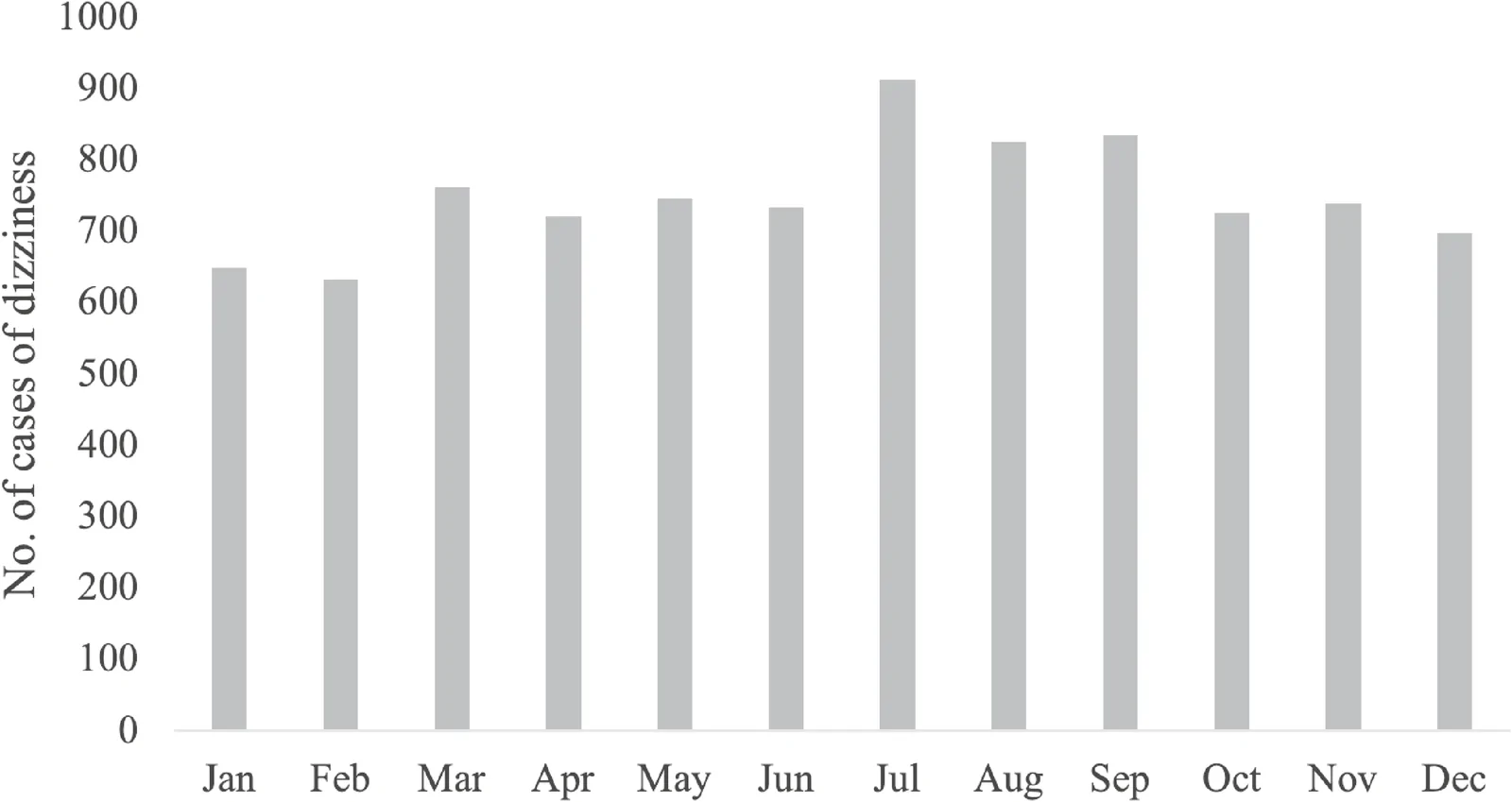
Monthly distribution of dizziness-related ambulance transports The bar chart shows the total number of dizziness-related ambulance transfers per month during the study period. Higher numbers were observed to occur during the summer months, peaking in July.

Table 1 summarizes the baseline characteristics of emergency ambulance transports according to dizziness status. Of the 270,120 eligible emergency transports, 8,981 (3.3%) patients were classified as dizziness-related ambulance transports. Patients with dizziness were older and more likely to be female than those without dizziness (*p* < 0.05 both). Dizziness-related ambulance transports occurred more frequently at home and were more commonly associated with mild injury severity. The seasonal distribution also differed significantly between the two groups.

**Table 1:** Baseline characteristics of patients who underwent dizziness-related ambulance transportation

	**All**	**Dizziness**	**No dizziness**	
	**270,120**	**8,981 (3.3)**	**261,139 (96.7)**	
	**n (%)**			***p***
Age				<0.05
0–29	28,241 (10.5)	205 (2.3)	28,036 (10.7)	
30–39	9,455 (3.5)	297 (3.3)	9,158 (3.5)	
40–49	13,770 (5.1)	587 (6.5)	13,183 (5.0)	
50–59	18,673 (6.9)	901 (10.0)	17,772 (6.8)	
60–69	31,241 (11.6)	1,622 (18.1)	29,619 (11.3)	
70–79	57,286 (21.2)	2,668 (29.7)	54,618 (20.9)	
≥80	111,454 (41.3)	2,701 (30.1)	108,753 (41.6)	
Sex				<0.05
Male	132,508 (49.1)	3,198 (35.6)	129,310 (49.5)	
Female	137,612 (50.9)	5,783 (64.4)	131,829 (50.5)	
Season				<0.05
Spring	63,706 (23.6)	2,229 (24.8)	61,477 (23.5)	
Summer	70,288 (26.0)	2,472 (27.5)	67,816 (26.0)	
Autumn	66,731 (24.7)	2,300 (25.6)	64,431 (24.7)	
Winter	69,395 (25.7)	1,980 (22.0)	67,415 (25.8)	
Place of occurrence				<0.05
Home	156,318 (57.9)	7,368 (82.0)	148,950 (57.0)	
Hospital	29,764 (11.0)	201 (2.2)	29,563 (11.3)	
Older adult facility	21,042 (7.8)	129 (1.4)	20,913 (8.0)	
Other	62,996 (23.3)	1,283 (14.3)	61,713 (23.6)	
Injury				<0.05
Mild	118,676 (43.9)	5,878 (65.4)	112,798 (43.2)	
Moderate	104,543 (38.7)	3,078 (34.3)	101,465 (38.9)	
Severe	40,537 (15.0)	15 (0.2)	40,522 (15.5)	
Death	4,534 (1.7)	0 (0.0)	4,534 (1.7)	
Other	1,830 (0.7)	10 (0.1)	1,820 (0.7)	

Values are presented as n (%). P-values were calculated using chi-square test.

Table 2 presents the results of the individual-level logistic regression analysis examining the associations between meteorological conditions and dizziness-related ambulance transports. In the crude analyses, higher mean air temperature and change in minimum temperature from the previous day were associated with slightly higher odds of dizziness-related ambulance transports; slightly lower odds appeared in association with a higher mean sea-level pressure, diurnal temperature range, change in sea-level pressure from the previous day, and longer sunshine duration. Total daily precipitation was also statistically associated with dizziness-related ambulance transports, albeit the association’s magnitude was small. No significant association was observed for changes in maximum temperature from the previous day.

**Table 2:** Associations between meteorological conditions and dizziness-related ambulance transports

**Variable**	**cOR**	**(95%CI)**	**aOR**	**(95%CI)**
Mean air temperature (°C)	1.010*	(1.007–1.013)	1.005*	(1.002–1.009)
Mean sea-level pressure (hPa)	0.990*	(0.987–0.993)	0.995*	(0.991–0.998)
Total daily precipitation (mm)	1.001*	(1.000–1.002)	1.001*	(1.000–1.002)
Sunshine duration (hours)	0.990*	(0.985–0.995)	0.991*	(0.986–0.996)
Change in maximum temperature from the previous day (°C)	0.996	(0.988–1.004)	0.995	(0.987–1.003)
Change in minimum temperature from the previous day (°C)	1.011*	(1.002–1.021)	1.010*	(1.000–1.020)
Diurnal temperature range (°C)	0.985*	(0.978–0.992)	0.992*	(0.985–0.999)
Change in sea-level pressure from the previous day (hPa)	0.992*	(0.987–0.997)	0.993*	(0.988–0.998)

cOR, crude odds ratio; aOR, adjusted odds ratio; CI, confidence interval.

The table reports the results of crude and adjusted logistic regression analyses. The adjusted models included age group, sex, season, place of occurrence, and injury status as covariates. *p < 0.05.

After adjustments for age group, sex, season, place of occurrence, and injury status, the observed associations remained largely unchanged. Mean air temperature (aOR 1.005, 95%CI 1.002–1.009) and change in minimum temperature from the previous day (aOR 1.010, 95%CI 1.000–1.020) were positively associated with dizziness-related ambulance transports. Meanwhile, mean sea-level pressure (aOR 0.995, 95%CI 0.991–0.998), sunshine duration (aOR 0.991, 95%CI 0.986–0.996), diurnal temperature range (aOR 0.992, 95%CI 0.985–0.999), and change in sea-level pressure from the previous day (aOR 0.993, 95%CI 0.988–0.998) were inversely associated with dizziness-related ambulance transport. Total daily precipitation remained significant (aOR 1.001, 95%CI 1.000–1.002) and the magnitude was minimal. Change in maximum temperature from the previous day was not significantly associated with dizziness-related ambulance transport. The magnitudes of the observed associations were generally small.

The number of dizziness-related ambulance transports per day ranged from 0 to 12, with a median of three transports per day. High-frequency days (≥5 dizziness-related ambulance transports per day) accounted for 26.9% of all days.

Table 3 presents the results of the logistic regression analysis examining the associations between meteorological conditions and high-frequency days for dizziness-related ambulance transports. In the primary analysis, higher mean air temperature was significantly associated with increased odds of a high-frequency day, whereas mean sea-level pressure showed an inverse association. Total daily precipitation was nonsignificantly associated with high-frequency days, while sunshine duration was non-significantly positively associated and the association was small. Sensitivity analyses using alternative definitions of high-frequency days (≥4 or ≥6 dizziness-related ambulance transports per day) showed associations for mean air temperature and mean sea-level pressure that were generally similar (in terms of direction and magnitude) to those observed in the primary analysis, whereas the associations for precipitation and sunshine duration varied across cutoff definitions.

**Table 3:** Associations between meteorological conditions and high-frequency days for dizziness-related ambulance transports

**Meteorological factors**	**≥5 dizziness-related ambulance transports per day**			

	**cOR**	**(95%CI)**	**aOR**	**(95%CI)**
Mean air temperature (°C)	1.031*	(1.019–1.043)	1.025*	(1.009–1.041)
Mean sea-level pressure (hPa)	0.973*	(0.960–0.986)	0.990	(0.972–1.008)
Total daily precipitation (mm)	1.002	(0.997–1.006)	1.000	(0.995–1.005)
Sunshine duration (hours)	1.000	(0.979–1.022)	1.005	(0.981–1.029)

Crude odds ratios (cORs) and adjusted odds ratios (aORs) with 95% confidence intervals were estimated using logistic regression models. The adjusted model included all meteorological variables shown in the table. Odds ratios represent the change in the odds per unit increase for each variable. A two-sided *p* < 0.05 was considered statistically significant. *p < 0.05.

Table 4 summarizes the associations between daily changes in meteorological conditions and high-frequency days for dizziness-related ambulance transports. In the adjusted analysis, an increase in maximum temperature from the previous day was significantly associated with higher odds of a high-frequency day (aOR 1.046, 95%CI 1.003–1.090), although the magnitude of the association was small. In contrast, changes in minimum temperature from the previous day were not significantly associated with high-frequency days (aOR 1.015, 95%CI 0.966–1.066). A smaller diurnal temperature range was significantly associated with an increased likelihood of a high-frequency day (aOR 0.944, 95%CI 0.907–0.982), whereas no significant association was observed for changes in sea-level pressure (aOR 1.006, 95%CI 0.983–1.031).

**Table 4:** Associations between daily meteorological changes and high-frequency days for dizziness-related ambulance transports

**Daily meteorological change variables**	**≥5 dizziness-related ambulance transports per day**			

	**cOR**	**(95%CI)**	**aOR**	**(95%CI)**
Change in maximum temperature from the previous day (°C)	1.017	(0.983–1.054)	1.046*	(1.003–1.090)
Change in minimum temperature from the previous day (°C)	1.036	(0.994–1.080)	1.015	(0.966–1.066)
Diurnal temperature range (°C)	0.961*	(0.931–0.993)	0.944*	(0.907–0.982)
Change in sea-level pressure from the previous day (hPa)	0.989	(0.970–1.009)	1.006	(0.983–1.031)

Crude odds ratios (cORs) and adjusted odds ratios (aORs) with 95% confidence intervals (CIs) were estimated using logistic regression models. The adjusted model included all meteorological variables shown in the table. Odds ratios represent the change in the odds per unit increase for each variable. A two-sided p-value of <0.05 was considered statistically significant. *p < 0.05.

The pairwise correlations among meteorological conditions were generally weak to moderate (all |r| < 0.50; Supplementary Table S1 in Additional file 1), and variance inflation factors were low (range: 1.39–1.55; Supplementary Table S2 in Additional file 1), suggesting that multicollinearity was unlikely to have materially influenced the regression estimates.

## Discussion

This population-based study focused on emergency transports showed that dizziness-related ambulance transports tended to be more frequent during the summer months, particularly in July. The associations observed for daily temperature changes differed somewhat between the individual- and day-level analyses. In the individual-level analysis (Table 2), an increase in minimum temperature from the previous day was associated with slightly higher odds of dizziness-related ambulance transports, whereas the association with an increase in maximum temperature was nonsignificant. In contrast, in the analysis of high-frequency days (Table 4), an increase in maximum temperature from the previous day was associated with higher odds of a high-frequency day, while the association with minimum temperature change was not significant.

A higher mean air temperature was associated with an increased likelihood of high-frequency days for dizziness-related ambulance transports, whereas the association with lower sea-level pressure was weaker and less consistent. In addition, short-term increases in maximum temperature and smaller diurnal temperature ranges were associated with high-frequency days for dizziness-related ambulance transports. Although there were modest absolute differences in meteorological variables, both sustained and short-term temperature-related conditions are shown here to be potentially associated with fluctuations in dizziness-related emergency demand. Acute increases in maximum temperature may impose thermoregulatory and cardiovascular strain, with the body often responding to this heat exposure through processes that facilitate heat dissipation, like peripheral vasodilation and sweating. However, these responses may also reduce central blood volume and alter cardiovascular regulation, potentially increasing susceptibility to dizziness or orthostatic symptoms [28, 29]. These physiological responses and the increase in susceptibility to dizziness may be particularly problematic in individuals with reduced cardiovascular or thermoregulatory reserve. Importantly, a smaller diurnal temperature range may reflect reduced within-day temperature variability, which in turn can represent persistently warm conditions or limited nocturnal cooling. Previous studies have reported associations between diurnal temperature range and various health outcomes, albeit the directions and magnitudes of these associations have varied across populations and outcomes [30]. It is worth mentioning that these mechanisms remain speculative and further studies incorporating individual-level information (e.g., hydration status, comorbidities, and actual indoor and outdoor temperature exposures) are needed to clarify them.

Our findings extend previous clinical and epidemiological knowledge on dizziness by demonstrating that meteorological conditions, particularly ambient temperature, are associated with population-level fluctuations in dizziness-related ambulance transports. A previous study conducted in Japan also reported significant associations between meteorological conditions and emergency transports for vertigo or dizziness. However, it was based on data from a single tertiary care hospital [23], whereas the present population-based analysis provides a more comprehensive assessment of real-world patterns, particularly in a regional setting with an aging population.

Dizziness encompasses a wide spectrum of peripheral and central etiologies with diverse pathophysiological mechanisms. Meanwhile, the variation across seasons observed in this study for dizziness-related ambulance transports, if coupled with the temperature-related associations, indicates that environmental conditions may be relevant to fluctuations in emergency medical service utilization for dizziness across multiple underlying conditions. What this entails is that environmental-related exacerbation may prompt emergency medical service utilization for dizziness-related cases. Importantly, the association observed with lower sea-level pressure was less consistent than that with temperature. Nevertheless, previous studies have suggested that changes in atmospheric pressure may affect vestibular function and trigger dizziness-related symptoms in susceptible individuals, although the underlying mechanisms remain incompletely understood. One plausible explanation for these findings is the increased occurrence of heat-related dizziness during elevated ambient temperature periods [31]. For instance, heat exposure can induce dehydration, peripheral vasodilation, and orthostatic hypotension, all of which may precipitate dizziness and result in emergency medical transport [32, 33]. In the context of the ongoing global warming phenomenon, summer heat has become more intense and prolonged in recent years, potentially amplifying these physiological stresses [34, 35]. Worthy of note is that older adults may be particularly vulnerable to heat-related dizziness [36], specifically because of age-related thermoregulation impairments and a reduced perception of heat stress [37, 38]. Previous studies have also pointed to the possibility that older individuals are more likely to refrain from using air conditioning even when available [39], particularly owing to economic concerns as well as behavioral and perceptual factors. Consequently, they may be at an increased risk of heat-related illness, including dizziness severe enough to require ambulance transport. These considerations combinedly suggest that rising temperatures associated with climate change may be related to seasonal increases in dizziness-related emergency demand. Further studies are warranted to better understand these associations and evaluate potential preventive approaches, particularly among older populations.

Although the study period overlapped with the COVID-19 pandemic, the year-to-year variation in dizziness-related ambulance transports was relatively modest. Consistent with previous population-based findings from Osaka Prefecture [40], the proportion of dizziness-related ambulance transports appeared largely stable during the pandemic. Therefore, the pandemic is unlikely to have substantially affected the overall temporal patterns observed in this study.

From a public health perspective, our results complement previous evidence linking short-term weather conditions to acute health events requiring emergency transportation, such as cerebrovascular diseases and cardiovascular emergencies [14–16, 22]. Unlike these conditions, dizziness is often perceived as benign, albeit it also frequently leads to emergency care because related symptoms are characterized by sudden onset, varying severity levels, and diagnostic uncertainty. The evidence in the current research underlines that short-term increases in temperature and reduced diurnal temperature variations may contribute to transient surges in dizziness-related emergency demand. What this brings forth is the need to consider meteorological conditions, particularly during warmer seasons, when planning emergency medical services and preventive strategies for vulnerable populations, including older adults.

A strength of our study was the use of a large dataset comprising more than 100,000 data points, including all emergency transport cases recorded in the Kochi-Iryo-Net database during the study period. This comprehensive population-based coverage minimizes selection bias, enabling a robust assessment of temporal patterns in dizziness-related ambulance transports. Another important strength was the integration of emergency medical service data with detailed meteorological information obtained from the Japan Meteorological Agency. By linking high-resolution weather data with daily ambulance transport records, we could evaluate both sustained and short-term meteorological influences on dizziness-related emergency demand in real-world conditions. This linkage enhanced the relevance of our findings for weather-informed public health planning and emergency medical service preparedness.

This study had several limitations. First, our analysis was restricted to dizziness-related cases that resulted in ambulance transport and, therefore, did not include individuals with mild or transient dizziness who did not seek emergency medical services. This means that the findings may not be fully representative of all dizziness episodes that occur in the community. However, because dizziness severe enough to prompt ambulance transport often reflects sudden onset, marked symptom severity, or diagnostic uncertainty, the captured cases are still likely to represent clinically and socially relevant events. Second, case identification was based on physician-recorded disease/injury names in the emergency transport database. The recorded terminology may have varied among physicians and institutions, and the database did not provide sufficient clinical detail to distinguish specific etiologies of dizziness or vertigo. Some degree of misclassification may have hence occurred, and the findings should be interpreted as reflecting dizziness-related ambulance transports in routine clinical practice, not specific vestibular or neurological disorders. Third, detailed individual-level clinical information, including medical history, comorbidities, and medication use, was not available in the emergency transport database. Since these factors may influence both dizziness occurrence and severity, as well as susceptibility to meteorological conditions, their absence may have resulted in residual confounding.

Fourth, this study was conducted using emergency transport data from Kochi Prefecture only, constraining the generalizability of the findings to other regions. Across regions, differences in short-term variations in temperature and atmospheric pressure, as well as geographical and climatic characteristics, may influence the relationship between meteorological conditions and dizziness-related emergency transports. Therefore, future multiregional or international studies would be valuable for assessing whether similar associations appear in different climatic and environmental contexts. Fifth, geographic discrepancies may have existed because meteorological data were obtained from only four observation stations, and these did not always correspond exactly to ambulance dispatch locations. The meteorological data in this study were assigned based on the nearest observation station to the fire department that received the emergency call; although ambulance services are typically provided by the nearest fire department to the site of occurrence, this approach still cannot secure the accuracy of local weather conditions at each ambulance transport location owing to the limited spatial coverage of monitoring sites. Furthermore, because detailed meteorological data were unavailable for some mountainous areas of Kochi Prefecture, the local weather conditions in these regions may not have been fully represented, and some degree of exposure misclassification may have occurred. Sixth, the meteorological variables were modeled as continuous variables and while assuming a linear association with the odds of high-frequency days for dizziness-related ambulance transports. This approach may not fully capture the potential nonlinear/threshold relationships between meteorological conditions and dizziness-related ambulance transports. Further studies using flexible nonlinear modeling approaches are warranted to investigate the shape and potential thresholds of these associations. Finally, information on patients who contacted emergency services but declined hospital transportation and detailed clinical outcomes after transport was unavailable. In addition, the dataset did not allow individual-level data linkage, making it impossible to identify repeated ambulance transports by the same individual.

## Conclusions

This population-based study found that dizziness-related ambulance transports tended to be more frequent during warmer months, particularly in July. Higher ambient temperatures and short-term temperature-related changes were associated with an increased likelihood of high-frequency days for dizziness-related emergency transports. In general, the findings suggest that meteorological conditions may be associated with temporal variations in dizziness-related ambulance transport demand. The observed associations were modest, the underlying mechanisms remain unclear, but still the results contribute to our understanding of how weather conditions may relate to patterns of emergency medical service utilization. Further studies incorporating more detailed clinical information are warranted to clarify the specific causes of dizziness underlying these associations and their potential implications for emergency medical service planning.

## Abbreviations

aOR: adjusted odds ratio
CI: confidence interval
cOR: crude odds ratio

## Supplementary information

**Additional file 1:** **Supplementary Table S1.** Pairwise correlation coefficients among meteorological conditions. **Supplementary Table S2.** Variance inflation factors for meteorological conditions.

**Additional file 2:** **Supplementary Figure S1.** Monthly trends in dizziness-related ambulance transports by year (2017–2023). Monthly numbers of dizziness-related ambulance transports in Kochi Prefecture are shown separately for each year from 2017 to 2023, illustrating the monthly temporal patterns within each study year.

## Declarations

### Availability of data and material

The data that support our findings are available from Kochi Prefecture (at marina.minami@kochi-u.ac.jp) upon reasonable request, but will not be made and are not publicly available.
