# Supplementary material for: Association between meteorological conditions and dizziness-related ambulance transports: a population-based study in Kochi Prefecture, Japan
Source: Environ Health Prev Med. 2026 Oct 20;31:71. doi: 10.1265/ehpm.26-00085 (PMC13719244; doi:10.1265/ehpm.26-00085)
Supplement: Supplementary file 1 [file ehpm-31-071-s001.docx]

**Additional file 1**

**Supplementary Table S1. Pairwise correlation coefficients among meteorological conditions**

| **Variable** | **Maximum temperature** | **Minimum temperature** | **Daily temperature range** | **Sea-level pressure** |
| --- | --- | --- | --- | --- |
| Maximum temperature | 1 | 0.169 | 0.443 | -0.139 |
| Minimum temperature | 0.169 | 1 | -0.252 | -0.482 |
| Daily temperature range | 0.443 | -0.252 | 1 | 0.287 |
| Sea-level pressure | -0.139 | -0.482 | 0.287 | 1 |

**Supplementary Table S2. Variance inflation factors for meteorological conditions**

| **Variable** | **VIF** |
| --- | --- |
| Daily temperature range | 1.55 |
| Maximum temperature | 1.44 |
| Sea-level pressure | 1.41 |
| Minimum temperature | 1.39 |
| Mean VIF | 1.45 |
